# Cytotoxic activity of Nep1‐like proteins on monocots

**DOI:** 10.1111/nph.18146

**Published:** 2022-05-03

**Authors:** Maikel B. F. Steentjes, Andrea L. Herrera Valderrama, Laetitia Fouillen, Delphine Bahammou, Thomas Leisen, Isabell Albert, Thorsten Nürnberger, Matthias Hahn, Sébastien Mongrand, Olga E. Scholten, Jan A. L. van Kan

**Affiliations:** ^1^ Laboratory of Phytopathology Wageningen University Wageningen 6708 PB the Netherlands; ^2^ Laboratoire de Biogènese Membranaire UMR 5200 CNRS University of Bordeaux F‐33140 Villenave d’Ornon France; ^3^ Department of Biology, Plant Pathology University of Kaiserslautern Kaiserslautern 67663 Germany; ^4^ Molecular Plant Physiology FAU Erlangen‐Nürnberg Erlangen 91058 Germany; ^5^ Center of Plant Molecular Biology University of Tübingen Tübingen 72076 Germany; ^6^ Plant Breeding Wageningen University & Research Wageningen 6708 PB the Netherlands

**Keywords:** *Botrytis squamosa*, cytotoxic activity, GIPC, Nep1‐like protein, onion (*Allium cepa*), phytotoxic protein, sphingolipids

## Abstract

- Necrosis‐ and ethylene‐inducing peptide 1 (Nep1)‐like proteins (NLPs) are found throughout several plant‐associated microbial taxa and are typically considered to possess cytolytic activity exclusively on dicot plant species. However, cytolytic NLPs are also produced by pathogens of monocot plants such as the onion (*Allium cepa*) pathogen *Botrytis squamosa*.
- We determined the cytotoxic activity of *B*. *squamosa Bs*Nep1, as well as other previously characterized NLPs, on various monocot plant species and assessed the plant plasma membrane components required for NLP sensitivity.
- Leaf infiltration of NLPs showed that onion cultivars are differentially sensitive to NLPs, and analysis of their sphingolipid content revealed that the GIPC series A : series B ratio did not correlate to NLP sensitivity. A tri‐hybrid population derived from a cross between onion and two wild relatives showed variation in NLP sensitivity within the population. We identified a quantitative trait locus (QTL) for NLP insensitivity that colocalized with a previously identified QTL for *B*. *squamosa* resistance and the segregating trait of NLP insensitivity correlated with the sphingolipid content.
- Our results demonstrate the cytotoxic activity of NLPs on several monocot plant species and legitimize their presence in monocot‐specific plant pathogens.

## Introduction

Microbial plant pathogens are dependent on their host as a source of nutrients that are required for proliferation and reproduction. Pathogens with a necrotrophic lifestyle, as well as hemibiotrophs that initially interact with their host as biotrophs but at a later stage of infection switch to necrotrophy, kill host cells to obtain nutrients and colonize host tissue. The killing of host cells is effectuated by the secretion of toxic molecules, referred to as effectors, that are able to induce host cell death responses. Some of the necrotic effector genes are uniquely present in certain pathogens and have host‐specific activity, while other effector genes are widely dispersed over several microbial taxa and are active in a broad range of hosts. One of the most notorious broad host range effectors is necrosis‐ and ethylene‐inducing peptide 1 (Nep1), which was originally identified in culture filtrates of *Fusarium oxysporum* pathogenic on coca plants. Infiltration of Nep1 into leaves of coca, as well as other dicot plant species, resulted in necrosis and ethylene production, which clarifies the etymology of Nep1 (Bailey, 1995). Since the discovery of Nep1, Nep1‐like proteins (NLPs) have been identified in many plant pathogenic fungi as well as other plant‐associated microbes such as bacteria and oomycetes (Seidl & Van den Ackerveken, 2019). Not all identified NLPs are able to induce necrosis, although the function of these proteins, classified as noncytolytic NLPs, remains to be discovered (Baxter *et al*., 2010; Dong *et al*., 2012; Lenarčič *et al*., 2019). All functionally characterized cytolytic NLPs, however, are able to induce necrosis upon leaf infiltration in all dicot plant species, but virtually no monocots (Gijzen & Nürnberger, 2006; Seidl & Van den Ackerveken, 2019).

The dicot‐specific activity of NLPs was resolved relatively recently by the elucidation of the NLP plant target (Lenarčič *et al*., 2017). The NLPs interact with glycosylinositol phosphorylceramide (GIPC), sphingolipids that are integral plant plasma membrane components located in the outer leaflet. More specifically, NLPs bind to the terminal hexose residues of GIPCs, which results in a conformational change that brings a loop with a hydrophobic amino acid residue in close contact with the plasma membrane. In *Arabidopsis thaliana*, NLPs were shown to oligomerize upon recruitment of an apoplastic leucine‐rich repeat protein, and the interaction of the plasma membrane and the loops of oligomerized NLPs is proposed to result in the formation of a pore with subsequent cytoplasmic leakage, ultimately causing cell death (Lenarčič *et al*., 2017; Chen *et al*., 2021). In dicot plants, GIPCs possess two terminal hexoses (series A), whereas in monocot plants they contain three hexoses (series B) (Cacas *et al*., 2013). Although NLPs can bind to GIPCs of both series A and B and in both cases undergo conformational change, the third hexose residue of series B GIPCs was proposed to create a physical distance between the protruded loop and the plasma membrane that prevents their interaction and the subsequent pore formation (Lenarčič *et al*., 2017).

Apart from their cytolytic activity, NLPs play an additional role in plant–pathogen interactions by triggering plant innate immune responses (Qutob *et al*., 2006). Leucine‐rich repeat receptor protein (LRR‐RP) RLP23 was shown to recognize NLPs and trigger immunity in *A*. *thaliana* (Albert *et al*., 2015). Both cytolytic and noncytolytic NLPs can trigger immunity, and the recognition is thus independent of the necrotic activity (Oome *et al*., 2014). Moreover, recognition did not require the full‐length protein, as a conserved fragment of only 20 amino acids found in most NLPs could trigger immunity as well (Böhm *et al*., 2014). Overexpression of RLP23 in potato conferred enhanced immunity against the NLP‐secreting pathogens *Phytophthora infestans* and *Sclerotinia sclerotiorum* (Albert *et al*., 2015).

Although the cytolytic activity of NLPs is typically considered to be exclusively effective on dicots, cytolytic NLPs also occur in the genomes of plant pathogens that are host‐specific on monocots. The lily (*Lilium*) pathogen *Botrytis elliptica*, for example, has two NLPs that could induce necrosis upon infiltration in dicot plants, but not in the monocot host plant lily (Staats *et al*., 2007a). The presence of NLPs with dicot‐specific cytolytic activity in microbial pathogens of monocot plants has for a long time been enigmatic. In this study, we aimed to analyze the biological activity of NLPs of the onion (*Allium cepa*) pathogen *Botrytis squamosa* and elucidate the role of cytolytic NLPs in the interaction between pathogens and monocot plants. *Botrytis squamosa* is the causal agent of onion leaf blight and is considered to use effector proteins to induce host cell death (Lorbeer *et al*., 2007; Steentjes *et al*., 2021a). Here, we report cytotoxic activity of NLPs on several monocot plant species, and demonstrate that different onion cultivars show differential sensitivity to NLPs, despite their similar GIPC series A : series B ratio. Furthermore, we were able to map a quantitative trait locus (QTL) for NLP insensitivity in an interspecific *Allium* tri‐hybrid population that segregates for resistance to *B*. *squamosa*.

## Materials and Methods

### Fungal strains, culture conditions and transformation procedure

*Botrytis squamosa* isolate MUCL31421 was used to inoculate onion leaves for RNA isolation and sequencing, and as recipient strain for transformation. Spores of *B*. *squamosa* were obtained by growth on autoclaved onion leaves on top of water agar and exposure to UV light as described in Steentjes *et al*. (2021b). For long‐term storage, spores were kept in 15% glycerol at −80°C. To obtain *B*. *squamosa BsNep1* knockout mutants, we used the CRISPR‐Cas9‐mediated protoplast transformation protocol optimized for *B*. *cinerea* as described by Leisen *et al*. (2020). Protoplasting conditions were optimized for *B*. *squamosa* by using 5 × 10^6^ spores to inoculate the liquid culture and allow growth for 24 h. Mutants were selected using an initial concentration of 17.5 µg ml^−1^ of hygromycin B. Knockout mutants were confirmed by PCR and sequencing of the gene fragment and obtained mutants were tested for their *in vitro* growth rate and ability to produce sclerotia and conidia.

### Plant material and NLP infiltration

**Fig. 1:**
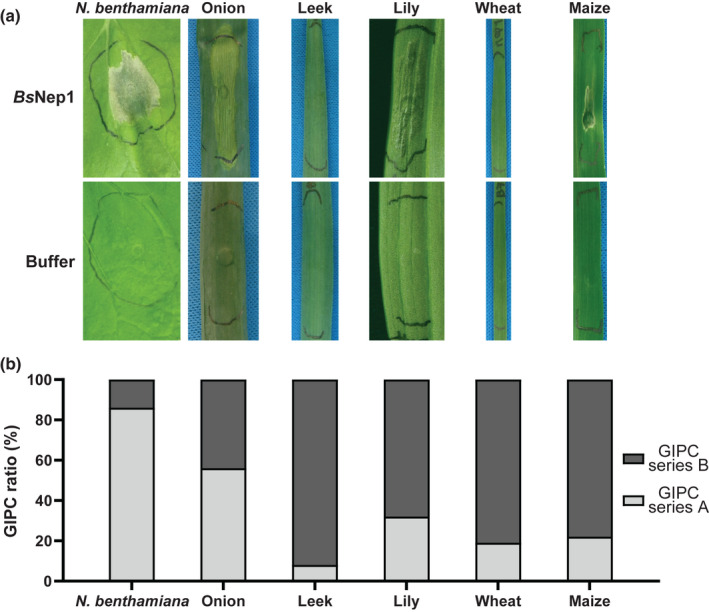
Plant response upon infiltration of *Botrytis squamosa* Nep1 and glycosylinositol phosphorylceramide (GIPC) composition. (a) Infiltration of *Bs*Nep1 and buffer into the dicot plant *Nicotiana benthamiana* and the monocots onion, leek, lily, wheat and maize. The infiltrated areas shown are representative of three replicate infiltrations that yielded similar plant responses and were assessed at 3 days post‐infiltration. (b) GIPC quantification of the infiltrated dicot and the monocot plants with series A representing two hexose moieties (Hex(R1)‐HexA‐IPC), and series B representing three hexose moieties (Hex‐Hex(R1)‐HexA‐IPC). Ratios are averages of three technical replicates.

**Fig. 2:**
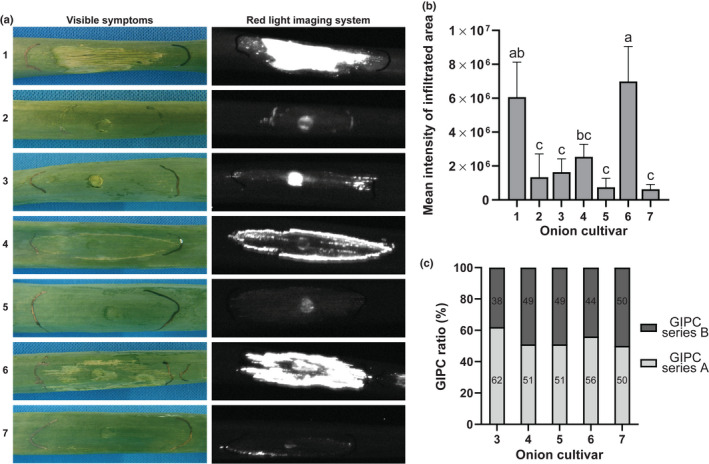
Responses of onion cultivars upon infiltration of *Botrytis squamosa* Nep1 in relation to their glycosylinositol phosphorylceramide (GIPC) content (a) Cell death response of seven onion cultivars upon infiltration of *Bs*Nep1. The left column shows visible necrotic symptoms and the right column shows the cell death intensity as observed by red light imaging. The infiltrated areas and red light images shown are representative of three replicate infiltrations and were assessed at 3 days post‐infiltration. (b) Quantification of cell death intensity of seven onion cultivars infiltrated with *Bs*Nep1 as measured by red light imaging at 3 days post‐infiltration. Values are averages of three replicates with subtracted background intensities and error bars represent the SD, with significant differences (*P* < 0.05) being indicated by different letters (Tukey *post hoc* analysis). (c) GIPC quantification of five onion cultivars, with series A representing two hexose moieties (Hex(R1)‐HexA‐IPC), and series B representing three hexose moieties (Hex‐Hex(R1)‐HexA‐IPC). Ratios are averages of three technical replicates.

**Fig. 3:**
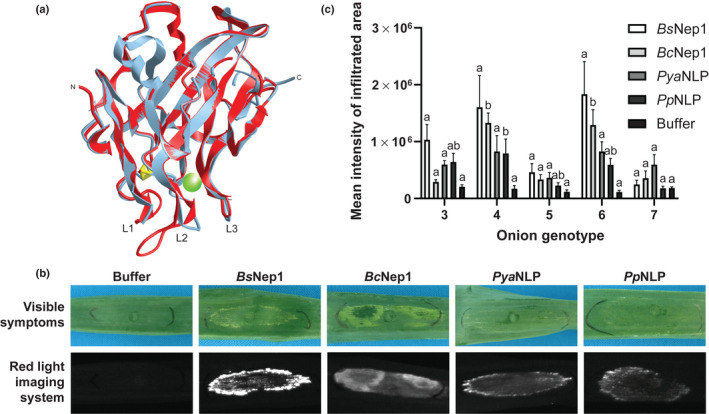
Comparing the cytotoxic activity of *Botrytis squamosa* Nep1 with other Nep1‐like proteins (NLPs). (a) Predicted protein structure of *Bs*Nep1 (red) using *Pya*NLP (gray) as a template. Loops 1–3 are marked at the lower part of the structures as L1, L2 and L3, and disulfide bonds are represented as yellow triangles. The proposed glycosylinositol phosphorylceramide (GIPC) binding site is indicated by a green sphere. (b) Cell death response of onion cultivar 6 upon infiltration of buffer and the four different NLPs, *Bs*Nep1, *Bc*Nep1, *Pya*NLP and *Pp*NLP. The upper row shows visible necrotic symptoms and the lower row shows the cell death intensity as observed by the red light imaging system. The infiltrated areas and red light images shown are representative of six replicate infiltrations and were assessed at 3 days post‐infiltration. (c) Quantification of cell death intensity of five onion cultivars (3–7) infiltrated with buffer and four different NLPs as measured by red light imaging at 3 days post‐infiltration. Values are averages of six replicates with subtracted background intensities and error bars represent the SE. Different letters per NLP indicate significant differences (*P* < 0.05) (Tukey *post hoc* analysis).

**Fig. 4:**
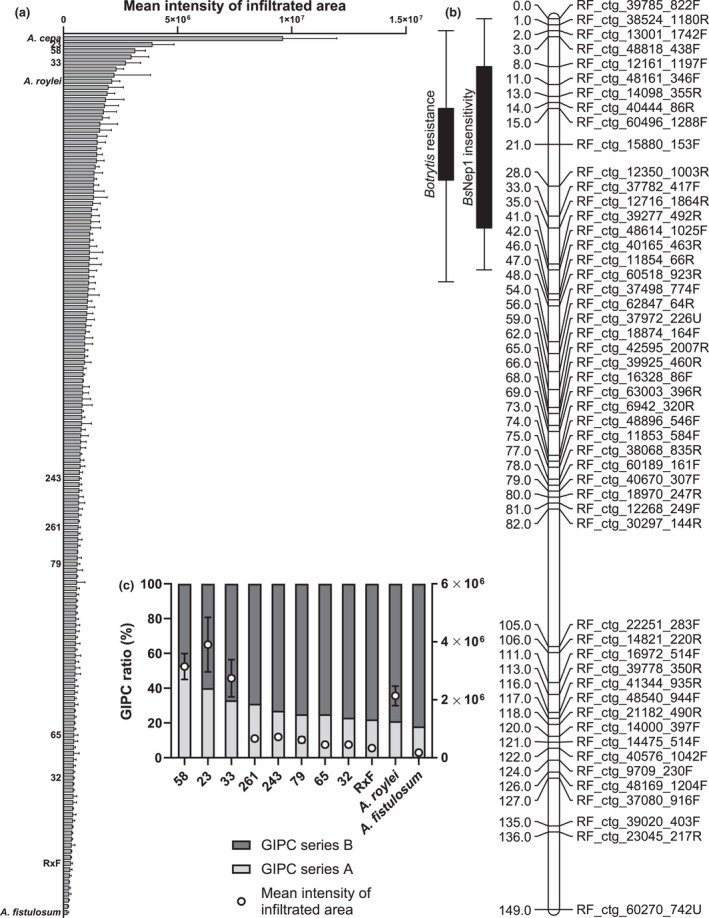
*Botrytis squamosa* Nep1 sensitivity and quantitative trait locus (QTL) mapping in an interspecific *Allium* tri‐hybrid population. (a) Cell death response of parental and progeny lines of the population (*A*. *cepa* × (*A*. *roylei* × *A*. *fistulosum*)) as measured by red light imaging at 3 days post‐infiltration. Values are averages of three replicates with subtracted background intensities and error bars represent the SE. Parental lines and progeny lines with quantified glycosylinositol phosphorylceramide (GIPC) content are indicated. (b) Genetic map of chromosome 6 of the interspecific tri‐hybrid population showing the newly identified QTL for *Bs*Nep1 insensitivity and the QTL for *B*. *squamosa* resistance. Lines show the logarithm of odds (LOD) region above the threshold value 3 and solid bars represent the 1 LOD interval from the maximum LOD score fitted within the significant region. (c) GIPC quantification and cell death response of a subset of parental and progeny lines, with series A representing two hexose moieties (Hex(R1)‐HexA‐IPC) and series B representing three hexose moieties (Hex‐Hex(R1)‐HexA‐IPC). Ratios are averages of three technical replicates. White circles represent the mean intensity of the infiltrated area as measured by red light imaging. Values are averages of three replicates with subtracted background intensities and error bars represent the SE.

Plant material used for NLP infiltration was as follows: *N*. *benthamiana* WT, *A. thaliana* Col‐0, maize (*Zea mays*) cv Golden Bantam, wheat (*Triticum aestivum*) cv Tadinia, leek (*Allium porrum*) cv Toledo, lily (*Lilium*) cv Asiatic, onion (*Allium cepa*) cv 1. Vuelta F1, 2. Musica F1, 3. Bruce F1, 4. Manesco F1, 5. Myskin F1, 6. Ceresco F1 and 7. Bonus F1. Plant materials from the *Allium* interspecific tri‐hybrid population (*A*. *cepa* × (*A*. *roylei* × *A*. *fistulosum*)) were obtained as described in Scholten *et al*. (2016). The population as well as the parental genotypes were maintained in tissue culture and plants of all genotypes were transferred to the glasshouse, transplanted in potting soil and grown for several weeks before infiltration. Plants were infiltrated using a syringe on the abaxial side of their leaves (where possible) with one of the four NLP proteins, *Bs*Nep1 (*Botrytis squamosa*), *Bc*Nep1 (*Botrytis cinerea*), *Pya*NLP (*Pythium aphanidermatum*) or *Pp*NLP (*Phytophthora parasitica*), or with buffer. NLP proteins were dissolved in 10 mM K_2_HPO_4_ buffer at a pH of 6.0. The final concentration of NLP proteins infiltrated in assays depicted in Figs 1 and 2 was 1 µM, while for assays depicted in Figs 3 and 4 and in Supporting Information Figs S4 and S7 the final concentration was 0.5 µM. Plant responses were assessed at 3 days post‐infiltration (dpi), either by evaluation of symptoms or by measuring cell death intensity as described in Villanueva *et al*. (2021). Synthesized peptides nlp20 (Böhm *et al*., 2014) and nlp27 from *Bs*Nep1 and *Bc*Nep1 (GIMYAWYFPKDQPAAGNVVGGHRHDWE) were both infiltrated in a final concentration of 1 µM at a pH of 6.0 and responses were observed at 3 dpi.

### Onion leaf infection assays and RNA isolation for expression analysis

For inoculation of *B*. *squamosa*, young but fully grown leaves of 2‐ to 4‐month‐old onion plants were used. For testing the virulence of the *BsNep1* knockout mutant, onion cultivars 1–7 were used, and for expression analysis, onion genotype DH was used. Detached leaves were placed in humid boxes and the cuticles of leaves were gently wiped with tissue paper to facilitate inoculation. Leaves were inoculated with 2 µl droplets of *B*. *squamosa* spores at 10^6^ spores ml^−1^ in 12 g l^−1^ potato dextrose broth. To assess the virulence, lesion sizes were measured at 3 dpi. For RNA isolation, plant tissues were sampled in three replicates at 16, 24 and 48 h post‐inoculation (hpi) and the inoculum used was sampled at 0 h. Samples were frozen in liquid nitrogen, freeze‐dried and ground to powder. For RNA extraction, samples were incubated in Trizol (Ambion; Life Technologies, Carlsbad, CA, USA) and treated with chloroform. Ethanol was added to the aqueous phase and the mixture was used to extract total RNA with the RNeasy Plant Mini Kit (Qiagen). This RNA was sent for sequencing. Reads were mapped to the annotated genome of *B*. *squamosa* (Valero‐Jiménez *et al*., 2020).

### Production and purification of *Bs*Nep1

The mature protein coding sequence of *Bs*Nep1 was amplified from cDNA and cloned into the vector pPIC9k (Invitrogen) via restriction enzymes *Eco*RI and *Not*I. After confirmation of the insert by PCR and sequencing, the plasmid was transformed into *Pichia pastoris* strain GS115 by electroporation. Fermentation was performed on a large scale using a BioFlo 120 fermentor (Eppendorf, Hamburg, Germany), applying the fermentation protocol described in Schouten *et al*. (2008). Briefly, *c*. 3 l of *P. pastoris* culture was grown at 30°C for 5 d. After 5 d the supernatant was separated by centrifugation and concentrated to 200 ml via diafiltration using a VivaFlow 200 Cassette MWCO 5000 Dalton (VF20P4; Sartorius, Göttingen, Germany). This concentrated supernatant was desalted and washed twice with 10 mM potassium phosphate at pH 7.0, and filter‐sterilized.

Protein purification was performed by ion exchange chromatography using Streamline SP XL (GE Healthcare, Uppsala, Sweden) at pH 7.0 as described in Schouten *et al*. (2008). Briefly, 5 ml of resin SP was equilibrated with 3 volumes of 10 mm KPO_4_, pH 7.0, in a glass Econo‐column (Bio‐Rad; 7 371 012) and transferred to a 50 ml tube containing 40 ml of concentrated crude protein sample. Proteins were allowed to bind by agitation on a horizontal rotor at 4°C for 90 min, and the glass column was filled again with the protein‐bound resin. The resin was rinsed by flushing the column twice with two column volumes of 10 mm KPO_4_, pH 7.0, and the bound proteins were recovered by eluting with an increasing salt concentration between 0.1 and 0.3 M NaCl in 10 mM K_2_HPO_4_. The eluted fractions were pooled, desalted and concentrated using Amicon Ultra‐15 PLBC filters. *Bs*Nep1 concentrations were quantified using Bradford assays (Bio‐Rad). Purified protein was analyzed by sodium dodecyl sulfate–polyacrylamide gel electrophoresis. *Bc*Nep1, *Pya*NLP and *Pp*NLP were produced and purified according to Schouten *et al*. (2008) and Lenarčič *et al*. (2017), respectively.

### GIPC composition analysis

Glycosylinositol phosphorylceramide analysis was performed by LC‐MS as described in (Cassim *et al*., 2021). Briefly, GIPCs were extracted according to Markham *et al*. (2006), incubated with methylamine solution to remove phospholipids and dried under air flow. Extracts were resuspended into 100 μl of THF/MeOH/H_2_O (40 : 20 : 40, v/v) with 0.1% formic acid containing the synthetic internal lipid standard C17 Glucosyl(β) Ceramide (d18 : 1/17 : 0) (Avanti Polar Lipids), thoroughly vortexed, incubated at 60°C for 20 min, sonicated for 2 min and transferred into LC vials. LC‐MS/MS analyses were performed in positive mode by multiple reaction monitoring (MRM) with a model QTRAP 6500 (ABSciex, Framingham, MA, USA) MS coupled to a LC system (1290 Infinity II; Agilent, Santa Clara, CA, USA). Reverse‐phase separations were performed at 40°C on a Supercolsil ABZ+, 100 × 2.1 mm column and 5 μm particles (Supelco; Sigma‐Aldrich). The areas of LC peaks were determined using MultiQuant software (v.3.0; ABSciex). For sphingolipid quantification, see Cassim *et al*. (2021). GIPC ratios are an average of three independent measurements.

### Protein sequence alignment and structure modelling

Mature protein sequences of *Bs*Nep1, *Bs*Nep2, *Bc*Nep1, *BcNep2* and *Pya*NLP were used as input for a Clustalw alignment and visualized using ESPript (Robert & Gouet, 2014). The tertiary structure of *Bs*Nep1 was predicted by Raptor X (Källberg *et al*., 2012). The 3GNZ protein model of *Pya*NLP was used as structure template for the prediction. Both models were compared using 3D‐Match (Softberry, Mount Kisco, NY, USA) and visualized using Cn3D (Wang *et al*., 2000).

### Reactive oxygen species measurements

Leaves of 4‐ to 6‐wk‐old *A. thaliana* Col‐0 and ΔRLP23 (Albert *et al*., 2015) were used to perform reactive oxygen species (ROS) measurements. Leaf discs were obtained using a disposable biopsy punch and were placed on water in a 96‐well plate and incubated overnight. After the water was removed, discs were treated with a 50 µl assay solution containing 10 µg ml^−1^ horseradish peroxidase (Sigma) and 50 µM luminol L‐012 (Fujifilm, Neuss, Germany). Immunity responses were triggered by the synthesized peptides nlp20 (1 μM) as described in Böhm *et al*. (2014), nlp27 (1 μM) from *Bs*Nep1 and *Bc*Nep1 (GIMYAWYFPKDQPAAGNVVGGHRHDWE), or with flg22 (0.1 μM) as a positive control or water as a negative control. Luminescence was measured by a microplate fluorescence reader (ClARIOstar; BMG Labtech) for 4 h.

### Statistical analysis

Statistical analysis of cell death intensity quantified by red light emission, as well as the virulence of the *BsNep1* knockout mutant, was performed in Spss Statistics 25.0 (IBM). To assess cell death intensities, a one‐way ANOVA with Tukey *post hoc* analysis for multiple comparisons was performed per NLP. To test the significance of the difference between the *BsNep1* knockout mutant and the wild‐type (WT), independent‐samples *t*‐tests were performed per cultivar. To assess differences between WT values, a one‐way ANOVA with Games–Howell *post hoc* analysis was performed. Differences were considered to be statistically significant with two‐tailed *P* values < 0.05. For the QTL mapping for NLP insensitivity, the analysis was performed in MapQTL 6 using interval mapping and mean values of red light emission per genotype as input, and the QTL was visualized on chromosome 6 using MapChart (Voorrips, 2002). Correlation analysis between GIPC series A content and NLP sensitivity of the genotypes of the tri‐hybrid population was performed in Spss Statistics 25.0 (IBM) using the nonparametric Spearman’s correlation coefficient.

## Results

### *Botrytis squamosa* Nep1 induces necrosis in a subset of monocot plant species

All species in the genus *Botrytis* are reported to possess two NLP genes (Staats *et al*., 2007b). In the genome of *B*. *squamosa* (Valero‐Jiménez *et al*., 2020), genes BSQU_015g03410 and BSQU_002g07590 encode orthologues of previously characterized proteins Nep1 and Nep2 from *B*. *cinerea* (Arenas *et al*., 2010). The protein sequences of Nep1 and Nep2 display 95% and 91% amino acid identity, respectively, between *B*. *squamosa* and *B*. *cinerea* (Fig. S1). Analysis of RNA‐sequencing data from *B*. *squamosa*‐infected onion leaves indicated that both genes are expressed during infection at different levels and with different temporal dynamics. *BsNep1* transcript levels gradually increased from the moment of inoculation until 24 hpi, and declined at 48 hpi. The *BsNep*2 transcript levels slightly increased at 16 hpi and then remained steady over the course of the experiment, but were always at lower levels than the *BsNep*1 transcript (Fig. S2).

We cloned the mature protein coding sequence of *BsNep1* in vector pPIC9k and produced the *Bs*Nep1 protein using the heterologous expression system *Pichia pastoris*. Ion‐exchange chromatography‐based purification yielded a protein of *c*. 25 kDa (Fig. S3). To evaluate its cytotoxic activity, *Bs*Nep1 was infiltrated into leaves of the dicot plant *Nicotiana benthamiana* which induced clear necrotic responses (Fig. 1a). Infiltration in leaves of the monocot plants onion, maize and lily, however, also resulted in necrotic leaf tissue, while leek and wheat did not show cell death responses upon infiltration of *Bs*Nep1 (Fig. 1a), indicating a differential response to *Bs*Nep1 between monocots. The intensity of the cell death reaction could not be quantified further due to the differences in leaf morphology between these phylogenetically distant plant species.

To examine a correlation between the variation in NLP sensitivity of monocot plant species and their membrane sphingolipid composition, we assessed the GIPC composition of a subset of monocot and dicot plants. The heretofore assumed dicot‐specific activity of NLPs was considered to rely on a difference in the ratio of terminal hexose residues of GIPCs between monocots and dicots. Dicots were shown to contain predominantly GIPCs with two hexose residues (series A) and monocots had a majority of GIPCs with three hexoses (series B), which structurally prevent NLPs from exerting their cytolytic activity (Cacas *et al*., 2013; Lenarčič *et al*., 2017). GIPCs were extracted from leaf material of NLP‐sensitive (onion, lily and maize) and ‐insensitive (wheat and leek) monocots and the dicot *N*. *benthamiana*, and the GIPC series A : series B ratio was determined using LC‐MS (Fig. 1b). Surprisingly, plant species with the same GIPC ratios can have different sensitivities to NLPs. For example, maize and wheat had contrasting NLP sensitivities but their GIPC ratios were highly similar with *c*. 20% series A GIPC. The NLP‐sensitive monocots onion and lily had relatively more series A GIPC compared with the NLP‐insensitive monocot leek, but their GIPC series A : series B ratio was still low compared with the dicot *N*. *benthamiana*.

### Onion cultivars are differentially sensitive to *Bs*Nep1 but have similar GIPC ratios

Because of the unexpected necrotic response of onion and other monocots upon infiltration of *Bs*Nep1, we verified the cytotoxic activity of *Bs*Nep1 on monocots by infiltrating a set of seven onion cultivars. A variation in response was observed between the cultivars ranging from strong necrosis in cultivars 1 and 6 to almost no visible symptoms in cultivars 5 and 7 (Fig. 2a). In order to objectively quantify the plant responses, we used a red light imaging system that allows quantification of cell death intensity of the infiltrated area (Villanueva *et al*., 2021). Cell death signal intensities were correlated with visual scoring of necrosis (Fig. 2a). Quantification of the red light emission yielded significant differences between onion cultivars that corresponded to the severity of visible symptoms (Fig. 2b).

To test whether the differences in NLP sensitivity between the onion cultivars were correlated with the GIPC composition, we assessed the GIPC composition of a subset of five onion cultivars. All analyzed cultivars showed a similar GIPC composition, with a GIPC series A : series B ratio varying between 50% and 62% (Fig. 2c). There was no correlation between GIPC ratios of the five onion cultivars and their sensitivity to NLPs, suggesting that monocot NLP sensitivity is not solely determined by the GIPC series A : series B ratio.

### Cytotoxic activity of NLPs on monocots is not specific for *Bs*Nep1

The cytotoxic activity of *Bs*Nep1 on a range of monocots evokes the hypothesis that *Bs*Nep1 functions in a different manner than previously characterized NLPs. Comparison of the protein structures of *Bs*Nep1 and the well‐characterized NLP of *Pythium aphanidermatum* (*Pya*NLP), of which the protein structure was resolved by crystallography (Ottmann *et al*., 2009), may provide some insight into the mechanisms underpinning the cytotoxic activity of *Bs*Nep1 on monocot plants. Aligning the amino acid sequences of *Bs*Nep1 and *Pya*NLP yielded a sequence identity of 43% (Fig. S1). The protein structure of *Bs*Nep1 was predicted using *Pya*NLP as a template. The modelled structure of *Bs*Nep1 very much resembled the structure of *Pya*NLP and contained identical loops and sheets (Fig. 3a). One substantial difference between the two protein structures is the elongation of loop 2 (L2) in *Bs*Nep1, which is a consequence of the insertion of the three amino acids N110, V111 and V112. Loop 3 (L3) is considered to be required for the interaction of NLPs with the plasma membrane and the subsequent insertion and pore formation. It could be hypothesized that the elongated L2 of *Bs*Nep1 may be able to take over, or accompany the function of L3, and bridge the physical distance between the NLP and the plasma membrane when bound to monocot GIPCs, and therefore effectuate cytotoxic activity on monocot plants.

To assess whether the predicted differences in protein structure between *Bs*Nep1 and *Pya*NLP lead to differential activity, we compared the necrotic effect of *Bs*Nep1 with the well‐characterized NLPs from *B*. *cinerea* (*Bc*Nep1), *Phytophthora parasitica* (*Pp*NLP) and PyaNLP in a set of monocot plant species. Surprisingly, all tested NLPs induced necrosis upon infiltration in onion (Fig. 3b). Moreover, in all monocot plant species in which *Bs*Nep1 induced necrosis (onion, maize and lily), all other NLPs were also active (Fig. S4), suggesting no difference in activity between *Bs*Nep1 and other NLPs. Furthermore, the ability of *Bs*Nep1 to trigger immunity by RLP23‐mediated recognition in *A*. *thaliana* was similar to other NLPs. Both the nlp20 peptide (derived from *Pya*NLP) and a corresponding *Bs*Nep1 peptide fragment of 27 amino acids (nlp27) were equally able to induce a ROS burst in *A. thaliana* WT but not in RLP23‐defective mutant lines (Fig. S5). In addition, the nlp20 and nlp27 peptides did not induce necrosis upon infiltration in *N*. *benthamiana* and *A. thaliana*, or in any of the seven tested onion cultivars (Fig. S6), indicating that the necrotic plant response upon infiltration of *Bs*Nep1 was caused by its cytotoxic activity and not its immunogenic patterns.

In addition to testing the cytotoxic activity of the four NLPs in monocot plants, we also infiltrated the proteins in a subset of five onion cultivars that previously showed variation in response to *Bs*Nep1 infiltration (Fig. S7). Quantification of the cell death intensity by red light imaging yielded differences in NLP sensitivity between onion cultivars (Fig. 3c). The differences in plant response had the same trend as observed upon infiltration of *Bs*Nep1, with cultivar 6 being relatively sensitive and cultivar 5 and 7 being relatively insensitive. Although there was variation in cell death intensity upon infiltration of different NLPs in the same onion cultivar, the differences in NLP sensitivity between cultivars were seemingly independent of which of the four NLPs was infiltrated.

### *Bs*Nep1 sensitivity segregates as quantitative trait in an interspecific *Allium* tri‐hybrid population

In view of the observed variation in sensitivity to *Bs*Nep1 between onion cultivars, we decided to test the sensitivity to *Bs*Nep1 in the tri‐hybrid population CCxRF that was derived from a cross between onion (*Allium cepa*) and an interspecific F_1_ hybrid (*Allium roylei* × *Allium fistulosum*). The CCxRF population segregates for resistance to *B*. *squamosa* with a QTL on chromosome 6 from *A*. *roylei* (Scholten *et al*., 2016). *Bs*Nep1 was infiltrated into leaves of 140 individual genotypes of the CCxRF population, as well as into the parental species *A*. *cepa*, *A. roylei* and *A. fistulosum*, and the hybrid plant *A*. *roylei* × *A*. *fistulosum* in three replicates. The response to the protein was quantified using the red light imaging system. Responses to *Bs*Nep1 infiltration varied between progeny lines of the population as well as between the parental genotypes, with the most intensive cell death observed for *A*. *cepa* and *A*. *roylei* and the least for *A*. *fistulosum* (Fig. 4a). The mean values of red light emission per genotype were used for QTL mapping. A QTL region originating from *A*. *fistulosum* was identified on chromosome 6. This region fully colocalized with the QTL region discovered earlier for resistance to *B*. *squamosa* from *A*. *roylei* (Fig. 4b).

To test whether the segregating trait of NLP sensitivity in the crossing population can be linked to the GIPC composition, we selected three parental lines and eight progeny lines for evaluation of their GIPC composition (Fig. 4c). Three offspring lines that were highly sensitive to *Bs*Nep1 (58, 23, 33) showed a relatively high GIPC series A : series B ratio, ranging from 33% to 53%. Three offspring lines with intermediate *Bs*Nep1 sensitivity (261, 243, 79) had a roughly similar and relatively low GIPC ratio, ranging from 25% to 31%, and two progeny lines with low *Bs*Nep1 sensitivity (65 and 32) had GIPC ratios of 23% and 25%, respectively. Parental lines RxF and *A*. *fistulosum* matched this pattern as they are both on the insensitive side of the *Bs*Nep1 sensitivity spectrum and showed low GIPC ratios (22% and 18%, respectively). By contrast, *A*. *roylei* is relatively sensitive to *Bs*Nep1 but has a low GIPC ratio of 21% and therefore deviates from the observed correlation between NLP sensitivity and GIPC composition in the crossing population. There was significant positive correlation between the NLP sensitivity and the proportion of series A GIPC with a correlation coefficient of *r* = 0.78 for all analyzed progeny and parental lines of the interspecific tri‐hybrid population (Fig. S8). Because of the deviating sensitivity of *A*. *roylei* to *Bs*Nep1, the correlation coefficient increased to *r* = 0.93 when analyzing exclusively progeny lines.

### *Bs*Nep1 does not contribute to virulence of *B. squamosa* on onion leaves

**Fig. 5:**
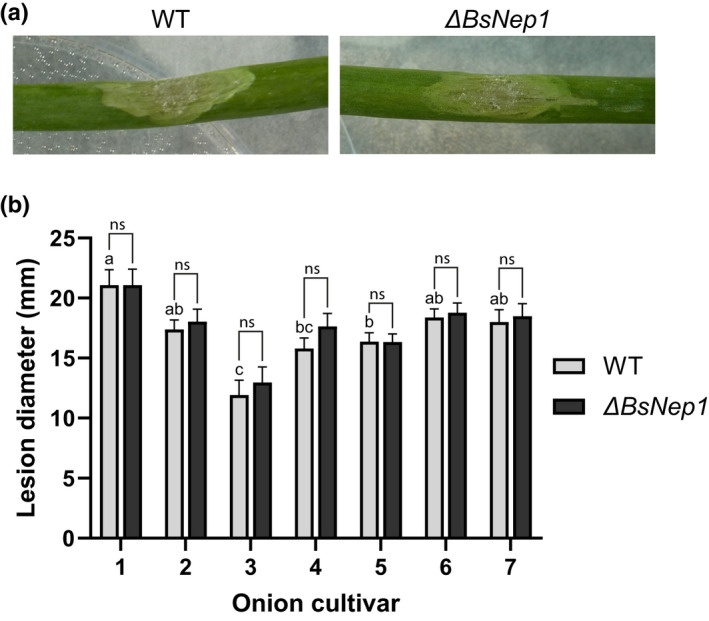
Virulence of *Botrytis squamosa* wild‐type (WT) and *BsNep1* knockout mutant on onion leaves. (a) Lesions of *B. squamosa* WT and *BsNep1* knockout mutant on onion cultivar 2 at 3 days post‐inoculation. (b) Virulence of WT and *BsNep1* knockout measured as lesion diameter on onion leaves at 3 days post‐inoculation. Error bars represent the SE with *n* = 35 per strain per cultivar (total *n* = 490). Differences between the WT and Δ*BsNep1* were assessed using an independent‐samples *t*‐test per onion cultivar and significant differences between WT values are indicated by different letters (*P* < 0.05; ns, not significant) (Games–Howell *post hoc* analysis).

In view of the cytotoxic activity of *Bs*Nep1 in onion leaves and the observation of cell death and tissue collapse during *B*. *squamosa* infection, we studied the contribution of *Bs*Nep1 to the induction of disease symptoms and the virulence of the fungus. Using the CRISPR‐Cas9‐mediated transformation protocol developed for *B. cinerea* (Leisen *et al*., 2020), we generated the first knockout mutant ever reported for *B*. *squamosa*. A homokaryotic mutant with the desired *BsNep1* deletion (Fig. S9) was assessed for its virulence as compared with the WT on leaves of seven different onion cultivars (Fig. 5a). No statistically significant differences in lesion size were observed between Δ*BsNep1* and the WT in any of the seven tested onion cultivars (Fig. 5b), suggesting that *BsNep1* does not play a role in the virulence of *B*. *squamosa* on onion leaves. Irrespective of the fungal strain, there were differences in *B*. *squamosa* susceptibility between the tested cultivars, with cultivar 1 being slightly more susceptible and cultivar 3 being slightly less susceptible than the other five cultivars, although in general all cultivars are highly susceptible to the pathogen. The difference in susceptibility between the cultivars does not correlate with their differential sensitivity towards *Bs*Nep1 (Fig. 2b), suggesting that NLP sensitivity is not required for susceptibility of onion to *B*. *squamosa*.

## Discussion

Since the first discovery of a necrosis‐ and ethylene‐inducing peptide 1 (Nep1) in 1995, many Nep1‐like proteins (NLPs) have been described in fungi, bacteria and oomycetes. To date, all functionally characterized cytolytic NLPs were reported to have an extremely broad host activity that is delimited to virtually only dicot plant species (Bailey, 1995; Seidl & Van den Ackerveken, 2019). One exception to this discrepancy is the ornamental plant species *Phalaenopsis amabilis*, a member of the monocot family Orchidaceae, which was shown to be sensitive to *Pya*NLP (Lenarčič *et al*., 2017). In this study, we show cytotoxic activity on a diverse set of monocot plant species for NLPs that were previously reported to be dicot‐specific. Among the five monocot species that were tested, three appeared to be sensitive to four distinct NLPs originating from fungi and oomycetes. It remains elusive as to why cytotoxic NLP activity has not been reported in more monocots in the 25 yr since the first functional analysis of an NLP. Studies that involve plant–microbe interaction on monocots often focus on cereal crop species such as rice and wheat, of which the latter was indeed found to be insensitive to NLPs in our study. By contrast, the related Poaceae crop species maize did develop necrosis upon NLP infiltration. Studies on the lily pathogen *Botrytis elliptica*, a close relative of *B*. *squamosa*, showed that NLPs of that species were not able to induce necrosis in lily (Staats *et al*., 2007a), whereas in the current study we observed lilies to be sensitive to NLPs. Possibly, the use of different lily cultivars explains the contrasting observations, analogous to the differences in NLP sensitivity between onion cultivars.

Surprisingly, we observed highly significant differences in GIPC composition between the different monocot species that could not be linked to their NLP sensitivity. Both maize and wheat had very similar GIPC ratios with low series A, whereas only maize was sensitive to NLPs. In addition, we observed that onion cultivars that varied in their sensitivity to NLPs all had a roughly similar GIPC series A : series B ratio of *c*. 50%. Altogether, these results suggest that the GIPC series A : series B ratio is not the sole determinant of NLP sensitivity of plant species.

Possibly, differences in leaf morphology such as leaf thickness or tissue structure may explain the observed variation in sensitivity between genotypes. To test whether the observed variation in NLP sensitivity depends on differences in tissue structure, or whether it is a characteristic of the membrane composition, sensitivity to NLPs could be assessed using protoplasts or plasma membrane vesicles (Ottmann *et al*., 2009).

An alternative explanation for the observation that the GIPC series A : series B ratio does not correlate with NLP sensitivity is that series A GIPCs consist of sphingolipids with different terminal sugar residues that bind NLPs with differential affinities (Cassim *et al*., 2021). If the terminal sugar residue composition of series A GIPCs differs between genotypes, then such genotype‐specific compositions may underlie the differential NLP sensitivity of onion cultivars or monocot plant species with equal series A : series B ratios.

In addition, the cytotoxic activity of NLPs on monocots might require the involvement of receptor proteins like the NTCD4 protein that was shown to aid in the oligomerization of NLPs and thereby promote cytolytic activity in *A. thaliana* (Chen *et al*., 2021). Such secondary target molecules could also determine the differences in NLP sensitivity observed between onion cultivars with similar GIPC compositions.

The GIPC ratio of *c*. 50% in onion cultivars differs from any other plant species for which the GIPC composition has been analyzed thus far, as dicots predominantly contain series A, while monocot GIPC composition is dominated by series B (Cacas *et al*., 2013; Lenarčič *et al*., 2017). We observed major differences in GIPC composition between the closely related species leek (*Allium porri*) and onion (*Allium cepa*) with series A : series B ratios of 10% and 50%, respectively. Moreover, progeny lines of the interspecific *Allium* tri‐hybrid population ranged in GIPC ratio between 20% and 50%. The observation that sphingolipid composition can vary between species of the same genus and their offspring was surprising, as essential cell membrane components such as sphingolipids were considered evolutionarily stable within phylogenetic lineages (Cacas *et al*., 2013).

The interspecific *Allium* tri‐hybrid population (*A*. *cepa* × (*A*. *roylei* × *A*. *fistulosum*)) showed variation in sensitivity upon infiltration of *Bs*Nep1. We identified a QTL for NLP insensitivity that colocalized with a previously identified QTL for *B*. *squamosa* resistance (Scholten *et al*., 2016). Moreover, the segregating trait of NLP sensitivity could be linked to the GIPC series A : series B ratio of the parental lines and progeny plants and showed a significant positive correlation between NLP sensitivity and the proportion of series A GIPC. The correlation was very high for the eight progeny lines tested (*r* = 0.93), but it might require more genotypes to provide a more robust statistical validation of this correlation. The QTL for NLP insensitivity present in *A*. *fistulosum* explained *c*. 16% of the phenotypic variation and colocalized with the previously identified QTL for *B*. *squamosa* leaf blight resistance present in *A*. *roylei* which explained 27–54% of the phenotypic variation in the same population (Scholten *et al*., 2016). It should be emphasized that all commercially available *A*. *cepa* cultivars are highly susceptible to *B*. *squamosa*, and the resistance QTL originating from *A*. *roylei* only confers partial resistance. The observation that both a disease resistance allele and a NLP insensitivity allele might originate from the same chromosomal location is enigmatic as both features have a different parental origin. The QTLs cover over 40 cM on the chromosome and are probably several hundreds of Mbp in length. It is clear that such a wide region may contain many genes. The onion genome size of 16 Gb (Finkers *et al*., 2021) and its biennial, self‐incompatible reproduction mode make further delineation and characterization of these QTLs a challenging task. It is conceivable that the NLP insensitivity and leaf blight resistance are separate traits that happen to be localized in a similar genomic region within the two parental *Allium* species. Whether or not the identified QTL for NLP insensitivity is based on genetic diversity in the regulatory or biosynthetic genes for GIPCs remains to be determined.

In this study, we did not obtain any evidence that *Bs*Nep1 contributes to virulence of *B*. *squamosa*. Knockout mutants of *Bs*Nep1 were not affected in lesion size as compared with the WT when inoculated on onion plants. Furthermore, cultivars that showed increased sensitivity to NLPs did not show increased disease development, indicating there is no correlation between NLP sensitivity and susceptibility to *B*. *squamosa* of onion cultivars. Earlier studies on NLPs of *B*. *cinerea* and *B*. *elliptica* showed that knockout mutants in either Nep1 or Nep2 did not display reduced virulence (Staats *et al*., 2007a; Arenas *et al*., 2010). The absence of one NLP gene in single knockout mutants could potentially be compensated by the other gene, although transcriptomic analysis showed that *B*. *squamosa* Nep1 is expressed to higher levels than Nep2. Moreover, upon infiltration, the cell death‐inducing activity of Nep1 from *B*. *cinerea* and *B*. *elliptica* was up to 10‐fold stronger than that of Nep2 (Staats *et al*., 2007a; Schouten *et al*., 2008). For *B*. *cinerea*, knockout mutants of multiple phytotoxic proteins, including Nep1 and Nep2, remained virulent on multiple host species, demonstrating the functional redundancy of virulence factors in *Botrytis* species (Leisen *et al*., 2022). For these reasons, we did not make an effort to generate a Nep1 Nep2 double mutant in *B*. *squamosa*, which is thus far more recalcitrant to transformation and targeted mutagenesis than *B*. *cinerea*.

Altogether, we show the functionality of cytotoxic NLPs on monocot plant species and legitimize the presence of cytotoxic NLPs in monocot‐specific pathogens. The identification of a QTL for NLP insensitivity that correlates with GIPC composition might help to track the genes involved in this trait and enable to further elucidate the sensitivity of plants to cytotoxic NLPs.

## Author contributions

MBFS, ALHV, OES and JALvK designed the research. MBFS and ALHV performed the experiments, LF, DB and SM performed sphingolipid extraction and analysis, TL and MH assisted in knockout mutant generation, and IA and TN provided NLP proteins. MBFS, ALHV and LF analyzed the data, and IA, TN, SM, OES and JALvK contributed to data interpretation. MBFS, OES and JALvK wrote the manuscript.

## Supporting information


**Fig. S1** Amino acid alignment of mature NLP proteins used in this study.
**Fig. S2** Expression levels of *Botrytis squamosa BsNep1* and *BsNep2* genes.
**Fig. S3** Protein gel of ion exchange chromatography‐based purification of *Bs*Nep1.
**Fig. S4** Infiltration of buffer and four different NLPs into dicot and monocot plants.
**Fig. S5** Reactive oxygen species assays in *Arabidopsis thaliana*.
**Fig. S6** Plant infiltrations of nlp20 and nlp27 peptides.
**Fig. S7** Cell death responses of onion cultivars 3–7 upon infiltration of buffer and four NLPs.
**Fig. S8** Correlation between GIPC ratio and *Bs*Nep1 sensitivity in the tri‐hybrid *Allium* population.
**Fig. S9** PCR amplicons confirming the deletion of the *BsNep1* gene.
Please note: Wiley Blackwell are not responsible for the content or functionality of any Supporting Information supplied by the authors. Any queries (other than missing material) should be directed to the *New Phytologist* Central Office.

## Data Availability

Data sharing is not applicable to this article as no datasets were generated or analyzed during the current study. The data that support the findings of this study are available from the corresponding author upon request.
