# Supplementary material for: Cytotoxic activity of Nep1‐like proteins on monocots
Source: New Phytol. 2022 May 3;235(2):690–700. doi: 10.1111/nph.18146 (PMC9320973; doi:10.1111/nph.18146)

New Phytologist

***New Phytologist* Supporting Information**

Article title: **Cytotoxic activity of Nep1-like proteins on monocots**
Authors: **Maikel B. F. Steentjes, Andrea L. Herrera Valderrama, Laetitia Fouillen, Delphine Bahammou, Thomas Leisen, Isabell Albert, Thorsten Nürnberger, Matthias Hahn, Sébastien Mongrand, Olga E. Scholten, Jan A. L. van Kan**
Article acceptance date: 30 March 2022

The following Supporting Information is available for this article:

Fig. S1 Amino acid alignment of mature NLP proteins used in this study.

Fig. S2 Expression levels of *B. squamosa BsNep1* and *BsNep2* genes.

Fig. S3 Protein gel of ion exchange chromatography-based purification of *Bs*Nep1.

Fig. S4 Infiltration of buffer and four different NLPs into dicot and monocot plants.

Fig. S5 Reactive oxygen species assays in *A. thaliana*.

Fig. S6 Plant infiltrations of nlp20 and nlp27 peptides.

Fig. S7 Cell death responses of onion cultivars 3-7 upon infiltration of buffer and four NLPs.

Fig. S8 Correlation between GIPC ratio and *Bs*Nep1 sensitivity in the tri-hybrid *Allium* population.

Fig. S9 PCR amplicons confirming the deletion of the *Bs*Nep1 gene.

Fig. S1 Amino acid alignment of mature proteins *Bs*Nep1, *Bs*Nep2, *Bc*Nep1, *Bc*Nep2 and *Pya*NLP. Amino acids highlighted in red indicate 100% identity between all proteins and blue squares indicate conservative replacement. Conserved cysteines are marked with asterisks and loops 1-3 are marked with black bars and labeled as L1, L2, and L3.

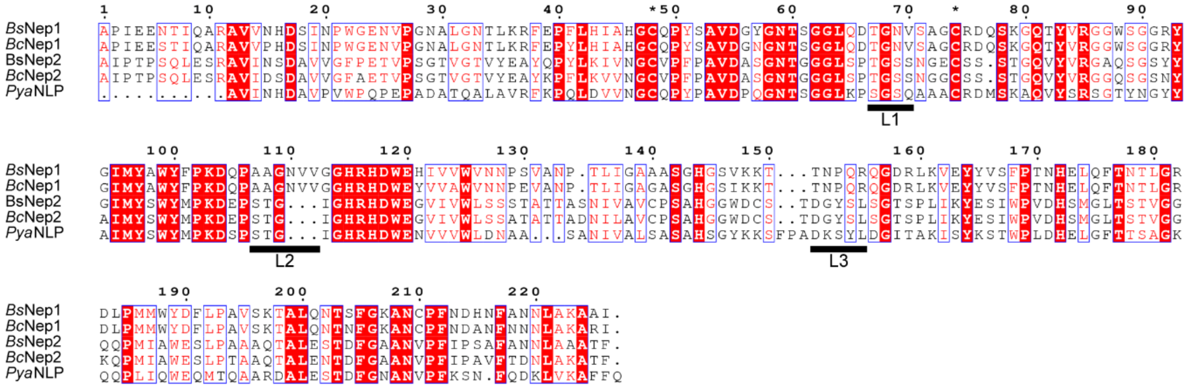

Fig. S2 Expression levels of *B. squamosa BsNep1* and *BsNep2* genes (in counts per million) in spores and during the infection of onion leaves at 16, 24 and 48 hours post inoculation (HPI). Values represent the average of three biological replicates, with error bars representing standard deviation.

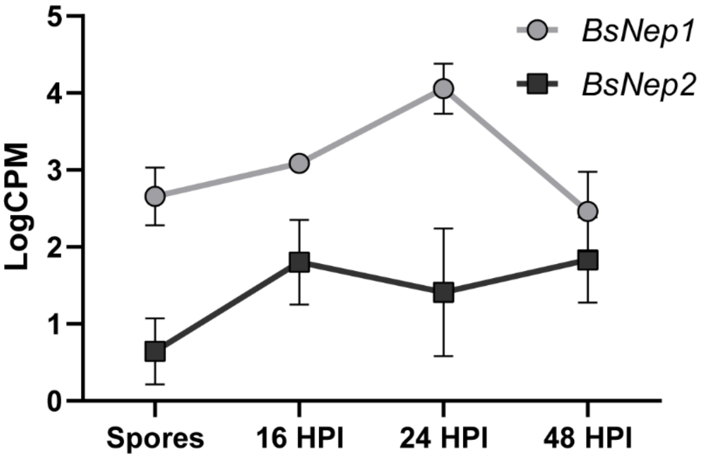

Fig. S3 Protein gel of ion exchange chromatography-based purification of *P. pastoris* produced *Bs*Nep1. Lanes 1-3 contain three elution fractions with purified *Bs*Nep1 protein with a size of ~25 kDa. A second protein that could not be eliminated in the purification is observed at ~15 kDa (M=marker).

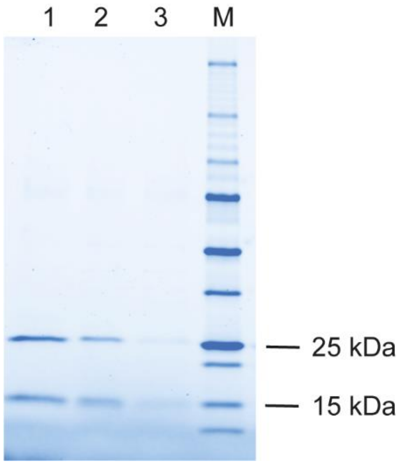

Fig. S4 Infiltration of buffer and the four different NLPs *Bs*Nep1, *Bc*Nep1, *Pya*NLP and *Pp*NLP into the dicot plants *Arabidopsis thaliana* and *Nicotiana benthamiana* and the monocots maize, leek, wheat and lily. Displayed infiltrated areas are a representative of three replicate infiltrations that yielded similar plant responses and were assessed at 3 days post infiltration.

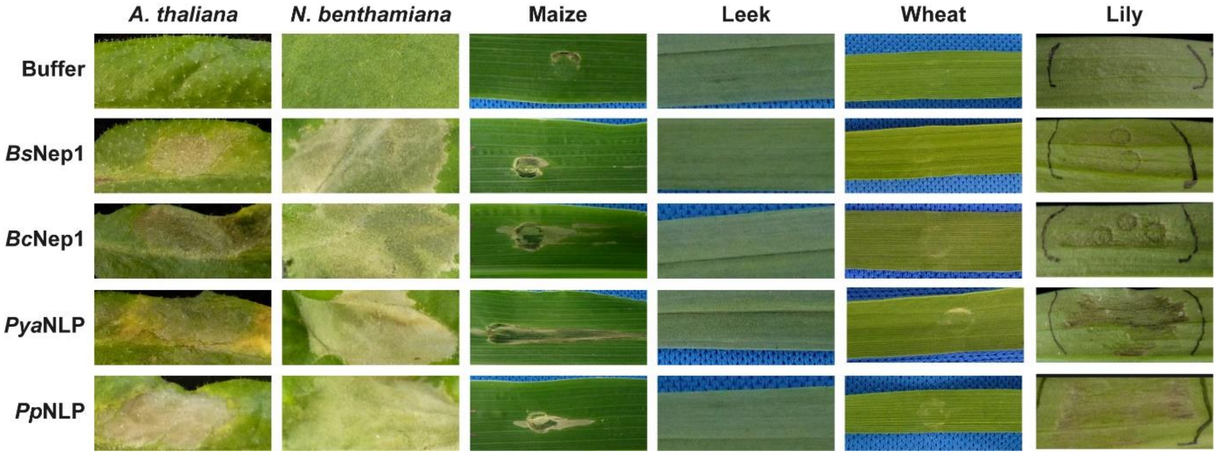

Fig. S5 Reactive oxygen species burst triggered by nlp20 (*PyaNLP*), nlp27 (*Bs*Nep1), flg22 as a positive control, and water as negative control in *Arabidopsis thaliana* Col-0 and rlp-23 defective mutant lines. Values are averages of measurements for 12 leaf disks per sample and error bars represent standard deviation.

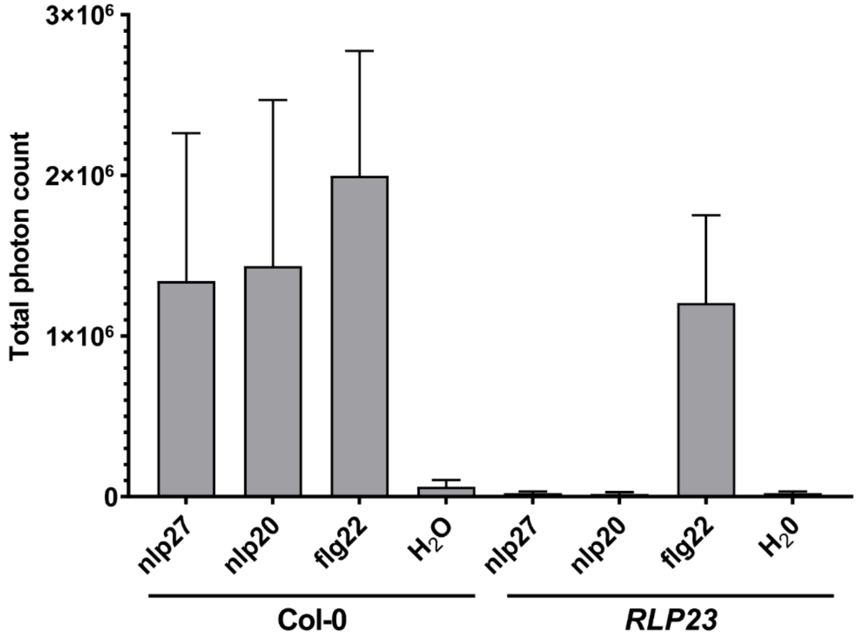

Fig. S6 Plant response of *Nicotiana benthamiana*, *Arabidopsis thaliana*, and onion cultivars 1-7 upon infiltration of 1µM nlp20 or nlp27 peptide. Displayed infiltrated areas were assessed at 3 days post infiltration.

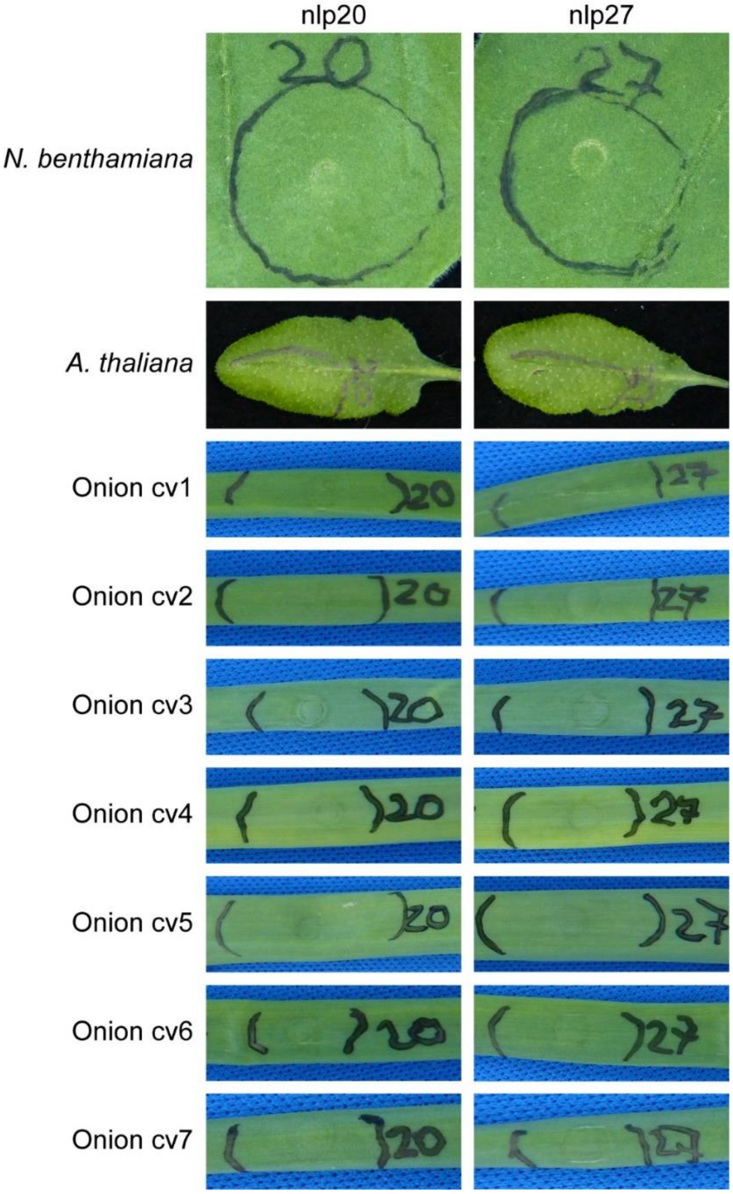

Fig. S7 Cell death responses of onion cultivars 3-7 upon infiltration of buffer and the four different NLPs *Bs*Nep1, *Bc*Nep1, *Pya*NLP and *Pp*NLP. Pictures on the left side show visible symptoms and on the right side show cell death intensity as observed by the red light imaging system. Displayed infiltrated areas and red light images are a representative of six replicate infiltrations and were assessed at 3 days post infiltration.

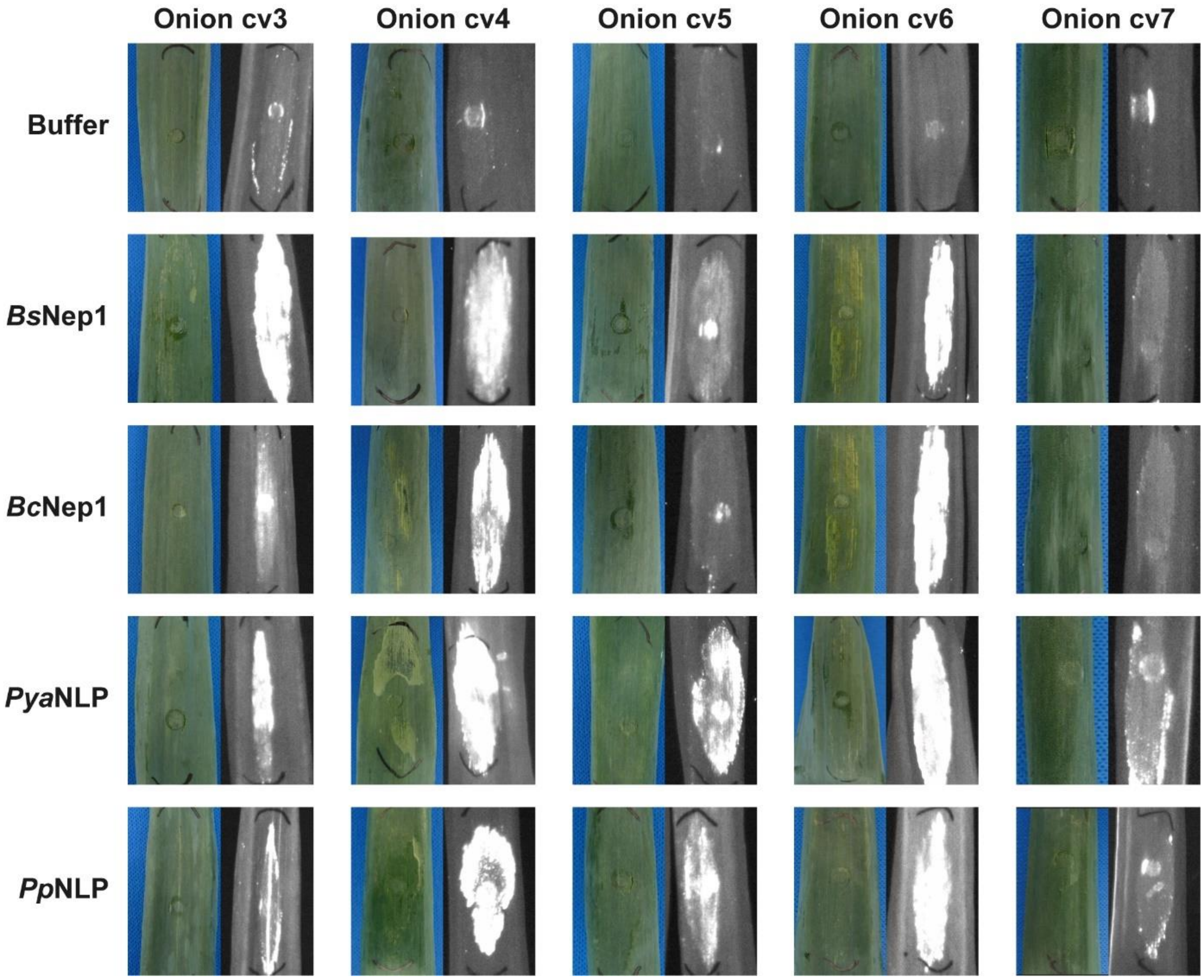

Fig. S8 Correlation between glycosylinositol phosphorylceramide (GIPC) ratio and *Bs*Nep1 sensitivity for a subset of progeny lines of the tri-hybrid *Allium* population as well as the parental lines *A. roylei*, *A. fistulosum* and the hybrid *A. roylei x A. fistulosum* (RxF). Spearman's correlation coefficient for all plant lines is r=0.782, when excluding *A. roylei* r=0.964 and when excluding all three parental lines r=0.929.

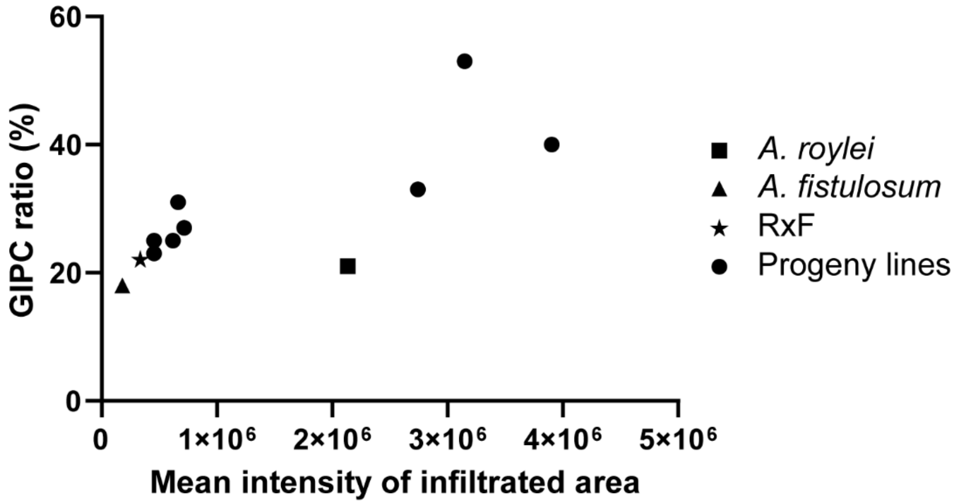

Fig. S9 PCR amplicons confirming the deletion of the *Bs*Nep1 gene. Primers were designed on the flanks of the deleted region. Bands appear on the expected amplicon size of 1221bp for wildtype (WT) and 2972bp for Δ*BsNep1*, indicating the successful replacement of the *Bs*Nep1 gene for the selection marker resistance cassette and confirming the homokaryosis of the deletion mutant.

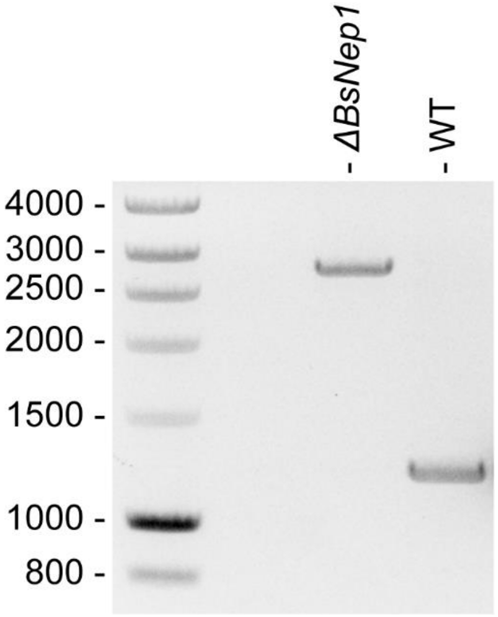

Supplement: Supplementary file 1 — Fig. S1 Amino acid alignment of mature NLP proteins used in this study. Fig. S2 Expression levels of Botrytis squamosa BsNep1 and BsNep2 genes. Fig. S3 Protein gel of ion exchange chromatography‐based purification of BsNep1. Fig. S4 Infiltration of buffer and four different NLPs into dicot and monocot plants. Fig. S5 Reactive oxygen species assays in Arabidopsis thaliana. Fig. S6 Plant infiltrations of nlp20 and nlp27 peptides. Fig. S7 Cell death responses of onion cultivars 3–7 upon infiltration of buffer and four NLPs. Fig. S8 Correlation between GIPC ratio and BsNep1 sensitivity in the tri‐hybrid Allium population. Fig. S9 PCR amplicons confirming the deletion of the BsNep1 gene. Please note: Wiley Blackwell are not responsible for the content or functionality of any Supporting Information supplied by the authors. Any queries (other than missing material) should be directed to the New Phytologist Central Office. [file NPH-235-690-s001.pdf]
